# Incidence of lingual nerve damage following surgical extraction of mandibular third molars with lingual flap retraction: A systematic review and meta-analysis

**DOI:** 10.1371/journal.pone.0282185

**Published:** 2023-02-27

**Authors:** Joshua Lee, Boxi Feng, Joon Soo Park, Magdalen Foo, Estie Kruger

**Affiliations:** 1 School of Allied Health, The University of Western Australia, Crawley, Australia; 2 International Research Collaborative - Oral Health and Equity, The University of Western Australia, Crawley, Australia; 3 Institute for Sustainable Industries & Liveable Cities, Victoria University, Melbourne, Australia; 4 UWA Dental School, The University of Western Australia, Nedlands, Australia; International Medical University, MALAYSIA

## Abstract

This systematic review and meta-analysis aimed to examine more recent data to determine the extent of lingual nerve injury (LNI) following the surgical extraction of mandibular third molars (M3M). A systematic search of three databases [PubMed, Web of Science and OVID] was conducted following the Preferred Reporting Items for Systematic Reviews and Meta-Analyses (PRISMA) guidelines. The inclusion criteria encompassed studies on patients who underwent surgical M3M extraction using the buccal approach without lingual flap retraction (BA-), buccal approach with lingual flap retraction (BA+), and lingual split technique (LS). The outcome measures expressed in LNI count were converted to risk ratios (RR). Twenty-seven studies were included in the systematic review, nine were eligible for meta-analysis. Combined RR for LNI (BA+ versus BA-) was 4.80 [95% Confidence Interval:3.28–7.02; P<0.00001]. The prevalence of permanent LNI following BA-, BA+ and LS (mean%±SD%) was 0.18±0.38, 0.07±0.21, and 0.28±0.48 respectively. This study concluded that there was an increased risk of temporary LNI following M3M surgical extractions using BA+ and LS. There was insufficient evidence to determine whether there is a significant advantage of BA+ or LS in reducing permanent LNI risk. Operators should use lingual retraction with caution due to the increased temporary LNI risk.

## Introduction

Lingual nerve injury (LNI) can have a detrimental effect on a patient’s quality of life (QoL) [1]. It can occur as a result of iatrogenic injury following oral and maxillofacial surgery (e.g. orthognathic surgery) [2] and the removal of mandibular third molars (M3M) [3]. LNI can lead to complications such as altered touch and taste sensation, neuralgia, as well as impaired swallowing and speech [4].

LNI following M3M removal is usually transient, with studies reporting the chance of spontaneous recovery to be 60% and 35% at three and six months respectively [4]. When LNI lasts more than six months, there is a significantly lower chance of spontaneous recovery and may be considered permanent [5]. Bagheri et al. found that patients with LNI lasting longer than nine months have less than 10% chance of recovery [4]. The incidence of permanent LNI following the removal of M3M is approximately 0.04–0.6% [6]. Though the incidence of LNI is low, the severity of the complications is deemed significant enough that patients should be informed of the risk of LNI before the procedure.

The lingual nerve (LN) is a branch of the mandibular division of the trigeminal nerve [7]. It provides somatosensory innervation such as pain, thermal, and pressure sensation to the mucous membrane. The LN innervates the anterior two-thirds of the tongue, lingual gingiva of the mandibular teeth, and the mucosa of the floor of the mouth [7]. Approximately 1 cm below where the inferior alveolar nerve and LN separates, the LN is joined by the chorda tympani nerve [8]. The path of the LN varies between individuals. A study by Pogrel et al. found that, in twenty cadaveric heads, the LN was in a range of 1 to 7 mm away from the lingual plate of the M3M [9]. This highlights the potential risk of LNI when a lingual flap is retracted due to varying locations of the LN.

The traditional approach to surgically removing the M3M is through raising a buccal flap and removing buccal bone, which can be done without (BA-) or with (BA+) lingual retraction [10]. An alternative approach is raising a lingual flap, placing a lingual retractor, and removing lingual bone, known as the lingual split technique (LS) [11]. The use of a lingual retractor has been associated with a higher incidence of LN paraesthesia [12]. The argument for this technique is that the incidence of recovery is higher, as severing the LN is avoided by protecting it with instruments and retractors. For example, the flap can be raised using Molt’s or Ward’s curved periosteal elevator and retracted using Walter’s lingual retractor [10]. Some also argue that lingual flap retraction provides improved access to the surgical site [10]. However, there are contrasting opinions. A systematic review, published in 2001, concluded that such retraction increased the tendency of temporary LNI [12]. However, this result was from analysing research performed pre-1999. Although there was a recent systematic review that was published [13], there is currently no published up-to-date study that quantitatively outlines the risks involved post-1999.

Therefore, this systematic review and meta-analysis aims to examine more recent data to determine the extent of LNI following the surgical removal of M3M. More specifically, comparing the incidence between three techniques: BA-, BA+, and LS.

## Materials and methods

### Ethical approval

The systematic review was registered on the PROSPERO database (Registration number: CRD42020181836).

### Study selection

The systematic review was registered on the PROSPERO database (Registration number: CRD42020181836) and conducted according to the Preferred Reporting Items for Systematic Reviews and Meta-Analyses (PRISMA) guidelines [14]. Two authors (J.L. and B.F.) independently conducted a systematic search of the literature in June 2022. The digital databases PubMed, Web of Science, and OVID were utilised to assess published studies that reported on the incidence of LNI following the surgical removal of M3M. Multiple searches were conducted using the following keyword combination: Firstly, “lingual nerve” AND buccal flap, retraction, oral surgery, third molar, lingual split. Secondly, “lingual nerve injury” AND buccal flap, retraction, oral surgery, third molar, lingual split. The connecting word ‘AND’ was used as a combination between the key words “lingual nerve” and “lingual nerve injury” and the rest of the search words. A limit was placed on the time of publication, with only articles published between May 1999 to June 2022 selected, as the previous landmark systematic review by Pichler and Beirne investigated studies from 1983 to May 1999 [12]. Grey literature was not assessed. All studies not in English were excluded. All studies except for systematic reviews were included. In the screening stage, the title and abstracts of publications were reviewed, and the duplicated studies were excluded. Subsequently, full-text copies were reviewed for eligibility for a systematic review. The inclusion criteria consisted of all studies on patients who have undergone surgical M3M extractions with BA-, BA+, or LS. Studies that did not meet the inclusion criteria were excluded. We utilised the software EndNote X9 (Clarivate, PA, USA) to organise the references and articles retrieved in the search. A consensus was reached after thorough discussion and gaining a third opinion from another author (J.P) if any discrepancies arose between the two examiners.

### Selection criteria

Population: The population for this systematic review is patients who underwent the surgical removal of M3M

Intervention/Comparison: The intervention was the extraction of M3M with or without lingual retraction or using the lingual split technique

Outcome: The outcome being measured within our study was the incidence of lingual nerve injury following the surgical extraction of M3M

### Search outcome

**Fig 1:**
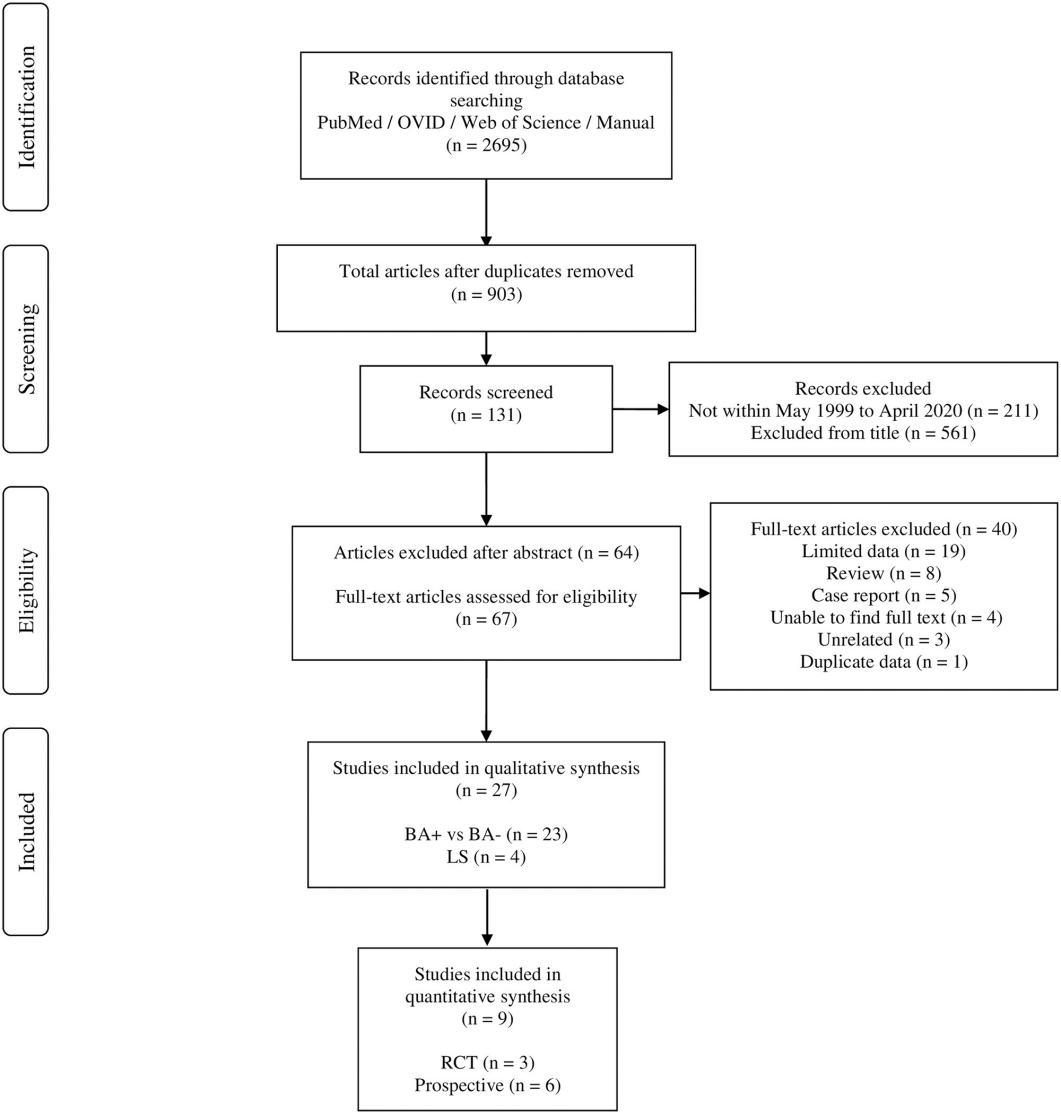
Process of data collection in accordance with the PRISMA statement for systematic reviews.

Fig 1 illustrates the outcomes of the search. From 2695 search results, 211 were excluded due to being outside the set time of publications, 1792 were removed due to being duplicates, 561 articles were excluded after analysing the title and a further 64 removed after analysing the abstract. Of the 67 potentially eligible studies, 40 citations were excluded. This left twenty-seven studies meeting the criteria and being suitable for inclusion in this systematic review [10, 15–40]. Of these, nine were suitable for quantitative synthesis and meta-analysis as information on LNI due to BA-, BA+, and LS was available [16, 17, 19, 21, 29, 32, 37, 38, 40].

### Data extraction

The following were extracted: study design, prospective/retrospective design, number of patients, number of teeth, age of patients, location of study, intervention, types of lingual retractors used, comparison, outcomes, and quantitative data, which was the rate of lingual nerve injury, both permanent and temporary. The primary outcome was LNI. Within LNI, prevalence of temporary and permanent LNI was also calculated.

### Data synthesis

The parameters for the meta-analysis consisted of analysing outcomes using the adopted risk ratio (RR) formula [41]:

$$\text{RR}=\left[\text{a}/{\text{n}}_{1}\right]/\left[\text{b}/{\text{n}}_{2}\right]$$

a = Number of LNI cases after BA+

b = Number of LNI cases BA-

n_1_ = Total number of cases after BA+

n_2_ = Total number cases after BA-

After obtaining the necessary parameters, a meta-analysis was undertaken in accordance with Review Manager software (RevMan 5.0 for Windows, Last Update—2014). The level of significance was set at 95% (P< 0.05). Statistical heterogeneity was determined based on the Tau-squared test, with a threshold P-value of 0.1 and inconsistency (I^2^) test value greater than 50% indicating high heterogeneity [42].

### Risk of bias

A critical appraisal tool to assess the quality of appropriate observation studies was utilised as per the checklist provided in the systematic review [43]. This included a series of questions with regards to the content of the Introduction, Methods, Results, Discussion, and Other sections. Furthermore, the cumulative count for these individual studies was tabulated.

### Inter-agreement reliability

Inter-agreement reliability was calculated between the two independent reviewers, in the data extraction (identification, screening, eligibility, and inclusion) stage (%). In addition, kappa statistics as a measure to test the inter-agreement reliability amongst the reviewers was computed during the risk of bias stage [SPSS version 27.0 (IBM Company, Chicago, Il, USA)] [44].

## Results

### Study characteristics and study results

Table 1 highlights the characteristics of all studies included in the systematic review. Except for six studies [22, 30, 32, 33, 36, 37], all the studies were prospectively designed. Five of the prospective studies were randomised controlled trials [21, 23, 29, 35, 38]. There was a total number of 24,985 participants, aged between 12–89 years old, with 32,866 teeth analysed. Studies were undertaken in Australia, Brazil, China, Greece, Hong Kong, India, Jordan, Nigeria, Pakistan, Singapore, Spain, United Kingdom, and the United States. There was a total number of 388 cases of temporary LNI in the systematic review. In many studies the use of a lingual retractor was at the clinician’s discretion and the type of lingual retractor used varied (Table 2). Table 3 highlights the prevalence of temporary LNI as outlined by all studies [10, 15–40]. Temporary LNI accounted for an average prevalence of 2.64%±2.25% standard deviation (SD). Table 4 highlights the prevalence of permanent LNI as outlined by all studies [10, 15–40]. Permanent LNI accounted for an average prevalence of 0.24%±0.41%SD.

**Table 1:** Characteristics of the studies which utilised BA-, BA+ and LS technique.

Author	Location	Prospective / Retrospective	Subjects	Teeth numbers	Age (Years)	Intervention	Comparison	Outcome (Primary and Secondary)	Quantitative data
Akadiri et al. 2009	Nigeria	Prospective	79	-	-	Three-sided buccal flap with buccal guttering BA-: 79	-	Incidence and duration of complicating nerve injuries	LNI (BA-) cases total: 3 (4.0%) LNI (Day 14) cases total: 1 (1%)
Baqain et al. 2010	Jordan	Prospective	321 M: 92 F: 229	443	Mean 22 Range: 16–66	Buccal mucoperiosteal flap BA-: 299 Buccal mucoperiosteal flap + lingual retraction using a Howarth periosteal elevator. BA+: 110 No data: 34	-	Presence of postoperative lingual neurosensory deficit	LNI (BA-) cases total: 1 LNI (BA+) cases total: 10 Recovery within 6 months: 11 Permanent cases total: 0 Lingual flap retraction: OR = 22.821 (95% Confidence Intervals: 2.83–183.83; p = 0.003)
Bataineh et al. 2001	Jordan	Prospective	741 M: 417 F: 324	741	<20: 61 20–30: 467 >30: 213	Buccal approach using a crestal incision extending to the distal of the second molar followed by a second incision extending downward and forward to the buccal sulcus BA-: 525 Buccal flap elevation + lingual retraction using a Howarth periosteal elevator BA+: 216	-	The rate and factors influencing sensory impairment of the inferior alveolar and lingual nerves after the removal of impacted mandibular third molars under local anaesthesia	LNI (BA-) cases total: 7 LNI (BA+) cases total: 12 Recovery within 12 months: 19 Permanent cases total: 0
Bataineh and Batarseh 2016	Jordan	Prospective	53 M: 23 F: 30	66	Mean: 23.3 Range: 16–43	Modified buccal flap without elevation of lingual flap BA-: 66	-	Evaluate a modified flap design for removal of lower third molars with avoidance of lingual flap elevation and its effect on postsurgical lingual nerve sensory impairment.	LNI cases total: 0
Charan babu et al. 2013	India	Prospective	100 M: 69 F: 31	100	16–25: 49 26–35: 36 36–45: 9 46–55: 4 55–65: 2	Moore/Gillbe collar technique after placement of Ward’s incision by a single oral surgeon BA-: 92 Moore/Gillbe collar technique after placement of Ward’s incision + lingual retraction with unspecified periosteal elevator BA+: 8	-	To evaluate the incidence and various risk factors influencing the sensory deficit in case of lingual nerve injury (LNI) in individuals whose impacted mandibular third molars are surgically removed under local anaesthesia.	LNI (BA-) cases total: 1 LNI (BA+) cases total: 3 Recovery within 3 months: 4 Permanent cases total: 0
Cheung et al. 2010	Hong Kong	Prospective	3595 M: 39% F: 61%	4388	Mean 27.2 Range 14–82	Extraction of 8s with or without raising of lingual flap Lingual flap raised: 1427 (33%) Attempt made to protect lingual tissue: 3477 (80%)	-	To determine the incidence of subsequent neurosensory deficit due to inferior alveolar nerve (IAN) and lingual nerve (LN) injury, to examine possible contributing risk factors and to describe the pattern of recovery	LNI cases total: 30 Stratification Without flap: 17 With flap: 13 Without retraction: 5 With retraction: 2 Recovery within the first 6 months: 15/26 Recovery after 24 months: 18/25 Permanent cases total: 7 (persist >2 years) (0.16%)
Gargallo-albiol et al. 2000	Spain	Prospective (RCT)	300 M: 140 F: 160	300 Totally impacted: 80 Partially erupted: 220	Mean: 27.4 Range: 14–59	Buccal mucoperiosteal flap BA-: 158 Buccal mucoperiosteal flap + lingual retraction with unspecified instrument BA+: 142	Comparison between protection of the lingual flap and without protection of the lingual flap	To evaluate the efficacy of protecting the lingual nerve by subperiosteal insertion of a retractor in 300 patients	LNI (BA-) cases total: 1 LNI (BA+) cases total: 3 Recovery after 21 days: 3 Recovery after 60 days: 1 Permanent cases total: 0
Ge et al. 2016	China	Retrospective	89 M: 46 F: 43	110 Deeply impacted: 47 Fully impacted: 63	Mean 33.2 Range: 22–56	Lingual split technique using piezosurgery all by the same surgeon under LA LS: 110	-	Primary outcome: Evaluate the effect and safety of lingual split technique using piezosurgery for the extraction of lingually positioned impacted mandibular 3rd molars Success rate, operating time (from the 1st incision to the last suture), and the incidence of major complications Secondary outcome: Pain, swelling, restricted mouth opening, and the postoperative symptom severity (PoSSe) score at the postoperative 7-day	LNI (LS) cases total: 6 (5.5%) Permanent cases total: 0
Gomes et al. 2005	Brazil	Prospective (RCT)	55	110	-	Buccal flap with a buccal retractor BA-: 55 Buccal flap with buccal retractor + lingual flap retraction using Free’s elevator BA+: 55 Patients were randomly allotted to have 1 side operated with buccal flap only and the other side with buccal and lingual flap retraction	Without lingual flap retraction Same patient, different technique for opposite side of mouth	Primary outcome: To clinically evaluate the frequency, type, and risk factors for lingual nerve damage after mandibular third molar surgery with reference to lingual flap retraction.	LNI (BA-) cases total: 0 LNI (BA+) cases total: 5 Recovered within 3 months: 5 Permanent cases total: 0
Janakiraman and Sanjay 2010	India	Prospective	119	119	Mean 27 Range: 18–35	Standard buccal Ward’s mucoperiosteal flap BA-: 6 Standard buccal Ward’s mucoperiosteal flap + lingual flap retraction with unspecified periosteal elevator BA+: 113	-	To determine the incidence of injury to the inferior alveolar and lingual nerves following surgical removal of impacted mandibular third molars and to evaluate the various factors contributing to the same.	LNI (BA-) cases total: 0 LNI (BA+) cases total: 5 Recovery in 6 months: 2 Still under observation (permanent): 1
Jerjes et al. 2006	United Kingdom	Prospective	1087 M: 505 F: 585	1087 Partially erupted: 857	Mean 23.3 Range 17–46	Envelope mucoperiosteal flap reflected and bone removal with a round bur in a straight hand- piece. Sectioning of tooth when needed. No lingual flap employed. BA-: 1087	-	The proportion of permanent sensory impairment of the inferior alveolar and lingual nerves and the factors influencing such prevalence after the removal of mandibular third molars under local anaesthesia.	LNI (BA-) cases total: 71 Permanent tongue paraesthesia 2 years after surgery: 11
Jerjes et al. 2010	United Kingdom	Prospective	3236 M: 1445 F: 1791	3236 Partially impacted: 2572 Close to IAC: 2531	Mean: 24.2 Range: 17–36 17–20: 852 21–25: 49.2 26–30: 471 >30: 319	An envelope mucoperiosteal flap reflected and bone was removed bucco-distally. No lingual flap employed. No lingual split technique used. BA-: 3236	-	Earlier reports, including a preliminary study within our unit, have shown that the surgeon’s experience is one of the most influential factors in determining the likelihood of both permanent inferior alveolar nerve (IAN) and lingual nerve (LN) paraesthesia, following third molar surgery. The effect of this and other factors influencing such prevalence are assessed in this study.	LNI (BA-) cases total: 57 Recovery after 6 months: 5 Recovery after 6–18 months: 15 Cases present 18–24 months after surgery: 37
Kale et al. 2014	-	Prospective	20 M: 13 F: 7	-	-	Standard Wards’ incision made in all cases. The tissue flap was reflected buccally, distally and lingually. The wide end of Howarth’s elevator was inserted adjacent to the lingual plate to protect the lingual nerve. Bone guttering on the buccal, distal and lingual side using straight fissure bur in low speed micrometre straight handpiece under copious irrigation. BA+: 20	-	To assess the clinical feasibility of lingual bone guttering technique for surgical extraction of mandibular third molars.	LNI (BA+) cases total: 0
Lata and Tiwari 2011	India	Prospective	90	-	-	Standard Terence Ward`s incision was made and after reflecting the buccal flap, a gutter in the disto-buccal bone was created. Bone removal done with motor-driven surgical bur under constant saline irrigation. Odontectomy or odontotomy procedure was done depending on the path of removal of the impacted tooth. No use of lingual retractor. BA-: 90	-	To determine the clinical incidence of lingual nerve injury following mandibular third molar removal and to analyse possible factors for the lingual nerve injury.	LNI (BA-) cases total: 6 LNI (6 months after surgery) cases total: 1
Majeed et al. 2018	Pakistan	Prospective (RCT)	300	300 Right: 170 Left:130	Range: 21–50 21–30: 245 31–40: 40 41–50: 15	BA-: 200 BA+: 100 No other information provided	-	To determine the incidence of lingual nerve injury and the effects of different variables on lingual nerve injury during mandibular third molars removal surgery.	LNI cases total: 18 LNI (Right): 6.47% LNI (Left): 5.38% LNI (BA-): 6 LNI (BA+): 12 Permanent LNI cases: Unclear. Author only stated, “there was a rapid improvement in the post-operative period”. This statement was interpreted as total permanent LNI cases = 0
Malden and Maidment 2002	United Kingdom	Retrospective	260	260	-	Mucoperiosteal flap raised buccal to the third molar. Distal relieving incision placed on retromolar pad to avoid all anatomical variants of the lingual nerve. Retracted with a Bowdler Henry rake retractor or Austin retractor. Lingual tissue retracted only to expose the occlusal aspect of the tooth or the superior aspect of mandibular bone covering the tooth or the crest of the lingual plate. No raising or elevating a lingual mucoperiosteal flap off the lingual aspect of the mandible. Tooth section with bone removal: 102 Bone removal without tooth section: 74 Soft tissue surgery: 84 BA-: 260	-	To determine if the incidence of lingual nerve damage differed to any significant extent from that reported elsewhere. To modify the operative technique, if indicated, to bring the incidence of nerve damage to within an accepted currently published standard.	LNI (BA-) cases total: 1 Permanent cases total: 0. Returned to full sensation within 6 weeks
Mavrodi et al. 2015	Greece	Prospective	-	1210 Left: 47.3% Right: 52.7%	Mean 48.5 Range: 15–82	Full thickness mucoperiosteal 3-cornered flap used in all cases 1. Classical bur technique: 470 2. Elevator placed on the buccal surface of the impacted molar to luxate the alveolar socket more easily: 740 Tooth sectioned 57.4% in group 1, 32.7% in group 2 BA-: 1210	-	To compare the efficacy and the postoperative complications of patients to whom two different surgical techniques were applied for impacted lower third molar extraction.	LNI cases total: 0
Moss and Wake 1999	United Kingdom	Retrospective	1614 M: 605 F: 1009	2906 Removed with a lingual flap: 2088	Range: 12–89	Buccal mucoperiosteal flap retraction BA-: 818 Buccal mucoperiosteal flap retraction + lingual flap retraction with the Hovell’s and Rowe retractors. BA+: 2088	-	To establish whether the deliberate raising of a lingual flap to allow the insertion of a broad lingual flap retractor in itself had any effect on lingual nerve morbidity.	LNI (BA-) cases total: 2 LNI (BA+) cases total: 16 Recovery within: 0–2 weeks: 8 3–6 weeks: 8 7–12 weeks: 1 13–25 weeks: 1 No permanent lingual sensory disturbance
Nguyen et al. 2014	Australia	Retrospective	6803	11599	-	Buccal flap with bone removal and tooth division BA-: 11599	-	To assess the incidence of and risk factors for permanent neuro- logic injuries to the inferior alveolar nerve (IAN) or lingual nerve (LN) after the removal of third molars.	LNI (BA-) cases total: 15 (0.15%) Temporary LNI cases total: 7 (0.069%) Permanent LNI cases total: 8 (0.079%)
Obiechina et al. 2001	Nigeria	Prospective	517 M: 297 F: 220	717	>16	Bur technique with preservation of lingual plate BA-: 699 Lingual bone split technique LS: 18	-	To analyse the depth of impaction of mandibular third molars, the type of anaesthesia, the surgical method used and the outcome.	LNI (BA-) cases total: 3 (0.4%) LNI (LS) cases total: 6 (0.8%) Complete recovery of lingual/labial sensation within 10–21 days Permanent case totals: 0
Pogrel and Goldman 2004	United States	Prospective	250	-	-	Buccal flap raised and an appropriate buccal retractor placed (usually Minnesota-type retractor). Lingual flap then raised using Molt or Ward’s periosteal elevator. Walter’s lingual retractor was then placed Lingual flap + buccal flap with a specially designed lingual retractor BA+: 250	-	The traditional approach in the United States has been a buccal approach avoiding exposure or surgery on the lingual side of the crest of the ridge. An alternative technique is to deliberately expose the lingual tissues and retract the lingual nerve lingually before tooth removal. This study reports a trial of this technique.	LNI (BA+) cases total: 4 Recovery within 21 days: 3 Recovery within 60 days: 1 Permanent cases total: 0
Praveen et al. 2007	India	Prospective (RCT)	90	90	Mean: 38 Range: 14–62	Buccal mucoperiosteal flap with buccal bone removal and tooth division + lingual nerve protection using Howarth’s periosteal elevator: BA+: 30 Buccal mucoperiosteal flap + lingual nerve protection using Howarth’s periosteal flap + normal/modified lingual split technique using a chisel LS: 60	-	To compare the morbidity rates of the three different surgical techniques and their efficacy with regard to postoperative pain, swelling, labial and lingual sensation.	LNI (BA+) cases total: 0 LNI (LS) cases total: 3 Recovery after 7 days: 1 Recovery after 14 days: 1 Permanent cases total: 1
Ramadorai et al. 2019	Singapore	Retrospective	1276 M: 458 F: 818	1276	Mean: 30.5 Range: 15–80	Buccal bone removal without raising a lingual flap BA-: 1276	-	To ascertain the incidence of IAN and LN damage after mandibular third molar surgery in National Dental Centre Singapore. Secondary outcome: To identify the contributory factors for the risk of IAN and LN nerve injury on the basis of the data collected.	LNI (BA-) cases total: 1 Recovery after 3 months: 1 Permanent cases total: 0
Robinson et al. 1999	United Kingdom	Retrospective	200	200	-	Buccal flap elevation without elevation of lingual mucoperiosteal flap: BA-: 110 Buccal flap elevation + Howarth periosteal elevator eased across the distal bone to the lingual side: BA+: 90	-	-	LNI (BA-) cases total: 1 LNI (BA+) cases total: 3 Complete recovery within 3 months Permanent cases total: 0
Shad et al. 2015	Pakistan	Prospective (RCT)	380 M: 179 F: 201	380	Mean: 25.6 Range: 18–38	Buccal flap elevation without elevation of lingual mucoperiosteal flap: BA-: 190 Buccal and lingual flap retraction + lingual flap retraction with Howarth’s periosteal elevator BA+: 190	-	-	LNI (BA-) cases total: 5 LNI (BA+) cases total: 17 Permanent cases total (BA-): 1 Although all showed signs of recovery within 3–6 months, 21 cases showed spontaneous recovery
Smith 2013	United Kingdom	Prospective	1000	1589	Mean 33.9 Range: 13–87	Buccal envelope mucoperiosteal flap. Lingual retraction was not used electively unless a significant amount of distal or distolingual bone removal was anticipated. BA-: 1455 LS: 134	-	To identify the relative risk of damage to the inferior dental (ID) and lingual nerves in patients undergoing lower third molar removal.	LNI (BA-) cases total: 3 LNI (LS) cases total: 2 Permanent cases total (BA-): 1 (0.06%)
Yadav et al. 2014	India	Prospective	1200	1200	Range: 18–45	Buccal mucoperiosteal flap BA-: 576 Buccal mucoperiosteal flap + lingual retraction with Howarth’s periosteal elevator BA+: 624	-	Investigate the incidence of sensory impairment of the lingual nerves following lower third molar removal and to compare the outcome with various operative variables.	1 week (Temporary) LNI (BA-) cases total: 10 LNI (BA+) cases total: 57 6 months (Permanent) LNI (BA-): 1 LNI (BA+): 3

BA+—Buccal approach with lingual flap retraction; BA-—Buccal approach without lingual flap retraction; F—Female; LNI—Lingual nerve injury; LS—Lingual split technique; M—Male; RCT—Randomised controlled trials

**Table 2:** Different types of lingual retractors used in M3M extraction.

Purpose Built	Non-purpose built	No Lingual Retraction	Not specified
Moss and Wake 1999	Baqain et al. 2010	Akadiri et al. 2009	Gargallo-albiol et al. 2000
Pogrel and Goldman 2004	Bataineh 2001	Bataineh and Batarseh 2016	Majeed et al. 2018
Smith 2013	Charan babu et al. 2013	Lata and Tiwari 2011	Obiechina et al. 2001
	Cheung et al. 2010	Jerjes et al. 2006	
	Ge et al. 2016	Jerjes et al. 2010	
	Gomes et al. 2005	Malden and Maidment 2002	
	Janakiraman and Sanjay 2010	Mavrodi et al. 2015	
	Kale et al. 2014	Nguyen et al. 2014	
	Praveen et al. 2007	Ramadorai et al. 2019	
	Robinson et al. 1999		
	Shad et al. 2015		
	Yadav et al. 2014		

Non-purpose built = Freer, Molt, Obwegesser, Howarth

Purpose built = Hovell ’s, Walter’s lingual retractor, Rowe

**Table 3:** Prevalence of temporary LNI using BA-, BA+ and LS.

	Total Teeth	Temporary LNI due to:			Total Temporary LNI	Prevalence (BA-)	Prevalence (BA+)	Prevalence (LS)	Total Prevalence
		BA-	BA+	LS					
Akadiri et al. 2009	79	3	N/A	N/A	3	3.80%	N/A	N/A	3.80%
Baqain et al. 2010	443	1	10	N/A	11	0.23%	2.26%	N/A	2.48%
Bataineh 2001	741	7	12	N/A	19	0.94%	1.62%	N/A	2.56%
Bataineh and Batarseh 2016	66	0	N/A	N/A	0	0.00%	N/A	N/A	0.00%
Charan babu et al. 2013	100	1	3	N/A	4	1.00%	3.00%	N/A	4.00%
Cheung et al. 2010	3595	U	U	N/A	30	U	U	N/A	0.83%
Gargallo-albiol et al. 2000	300	1	3	N/A	4	0.33%	1.00%	N/A	1.33%
Ge et al. 2016	110	N/A	N/A	6	6	N/A	N/A	5.45%	5.45%
Gomes et al. 2005	110	0	5	N/A	5	0.00%	4.55%	N/A	4.55%
Janakiraman and Sanjay 2010	119	0	5	N/A	5	0.00%	4.20%	N/A	4.20%
Jerjes et al. 2006	1087	71	N/A	N/A	71	6.53%	N/A	N/A	6.53%
Jerjes et al. 2010	3236	57	N/A	N/A	57	1.76%	N/A	N/A	1.76%
Kale et al. 2014	20	N/A	0	N/A	0	N/A	0%	N/A	0.00%
Lata and Tiwari 2011	90	6	N/A	N/A	6	6.67%	N/A	N/A	6.67%
Majeed et al. 2018	300	6	12	N/A	18	2.00%	4.00%	N/A	6.00%
Malden and Maidment 2002	260	1	N/A	N/A	1	0.38%	N/A	N/A	0.38%
Mavrodi et al. 2015	1210	0	N/A	N/A	0	0.00%	N/A	N/A	0.00%
Moss and Wake 1999	2906	2	16	N/A	18	0.07%	0.55%	N/A	0.62%
Nguyen et al. 2014	11599	15	N/A	N/A	15	0.13%	N/A	N/A	0.13%
Obiechina et al. 2001	717	3	N/A	6	9	0.42%	N/A	0.84%	1.26%
Pogrel and Goldman 2004	250	N/A	4	N/A	4	N/A	1.60%	N/A	1.60%
Praveen et al. 2007	90	N/A	0	3	3	N/A	0.00%	3.33%	3.33%
Ramadorai et al. 2019	1276	1	N/A	N/A	1	0.08%	N/A	N/A	0.08%
Robinson et al. 1999	200	1	3	N/A	4	0.50%	1.50%	N/A	2.00%
Shad et al. 2015	380	5	17	N/A	22	1.32%	4.47%	N/A	5.79%
Smith 2013	1589	3	N/A	2	5	0.19%	N/A	0.13%	0.31%
Yadav et al. 2014	1200	10	57	N/A	67	0.83%	4.75%	N/A	5.58%
					388	Mean—1.24% ±1.91%SD	Mean—2.39% ±1.68%SD	Mean—2.44% ±2.11%SD	Mean—2.64% ±2.25%SD

SD—Standard deviation; N/A—Not applicable; U—Unspecified

**Table 4:** Prevalence of permanent LNI using BA-, BA+ and LS.

	Total Teeth	Permanent LNI due to:			Total Permanent LNI	Prevalence (BA-)	Prevalence (BA+)	Prevalence (LS)	Total Prevalence
		BA-	BA+	LS					
Akadiri et al. 2009	79	U	N/A	N/A	U (No follow up after 14 days)	U	N/A	N/A	U
Baqain et al. 2010	443	0	0	N/A	0	0.00%	0.00%	N/A	0.00%
Bataineh 2001	741	0	0	N/A	0	0.00%	0.00%	N/A	0.00%
Bataineh and Batarseh 2016	66	0	N/A	N/A	0	0.00%	N/A	N/A	0.00%
Charan babu et al. 2013	100	0	0	N/A	0	0.00%	0.00%	N/A	0.00%
Cheung et al. 2010	3595	U	U	N/A	7	U	U	N/A	0.19%
Gargallo-albiol et al. 2000	300	0	0	N/A	0	0.00%	0.00%	N/A	0.00%
Ge et al. 2016	110	N/A	N/A	0	0	N/A	N/A	0.00%	0.00%
Gomes et al. 2005	110	0	0	N/A	0	0.00%	0.00%	N/A	0.00%
Janakiraman and Sanjay 2010	119	0	1	N/A	1	0.00%	0.84%	N/A	0.84%
Jerjes et al. 2006	1087	11	N/A	N/A	11	1.01%	N/A	N/A	1.01%
Jerjes et al. 2010	3236	37	N/A	N/A	37	1.14%	N/A	N/A	1.14%
Kale et al. 2014	20	N/A	0	N/A	0	N/A	0.00%	N/A	0.00%
Lata and Tiwari 2011	90	1	N/A	N/A	1	1.11%	N/A	N/A	1.11%
Majeed et al. 2018	300	0	0	N/A	0	0.00%	0.00%	N/A	0.00%
Malden and Maidment 2002	260	0	N/A	N/A	0	0.00%	N/A	N/A	0.00%
Mavrodi et al. 2015	1210	0	N/A	N/A	0	0.0%	N/A	N/A	0.00%
Moss and Wake 1999	2906	0	0	N/A	0	0.00%	0.00%	N/A	0.00%
Nguyen et al. 2014	11599	8	N/A	N/A	8	0.07%	N/A	N/A	0.07%
Obiechina et al. 2001	717	0	N/A	0	0	0.00%	0.00%	0.00%	0.00%
Pogrel and Goldman 2004	250	N/A	0	N/A	0	N/A	0.00%	N/A	0.00%
Praveen et al. 2007	90	N/A	0	1	1	N/A	0.00%	1.11%	1.11%
Ramadorai et al. 2019	1276	0	N/A	N/A	0	0.00%	N/A	N/A	0.00%
Robinson et al. 1999	200	0	0	N/A	0	0.00%	0.00%	N/A	0.00%
Shad et al. 2015	380	1	0	N/A	1	0.26%	0.00%	N/A	0.26%
Smith 2013	1589	1	N/A	0	1	0.06%	N/A	0.00%	0.06%
Yadav et al. 2014	1200	1	3	N/A	4	0.08%	0.25%	N/A	0.33%
					72	Mean—0.18% ±0.38%SD	Mean—0.07% ±0.21%SD	Mean—0.28% ±0.48%SD	Mean—0.24% ±0.41%SD

SD—Standard deviation; N/A—Not applicable; U—Unspecified

### Meta-analysis

**Fig 2:**
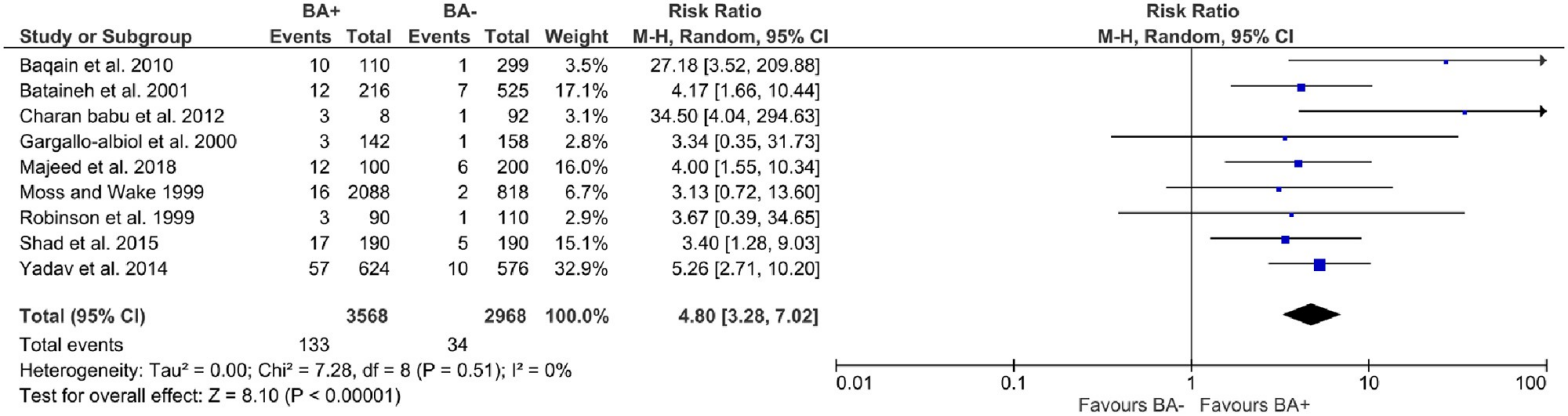
Forest plot outlining the risk of lingual nerve injury based on prospective clinical trials.

All studies that were included in the meta-analysis are outlined in Fig 2. Comparing BA+ vs BA-, the overall RR was 4.80 [95% Confidence Interval: 3.28–7.02; P < 0.00001], with a range of 3.13 to 34.50, and with negligible evidence of heterogeneity (I^2^ = 0%).

### Risk of bias

Table 5 outlines the assessment of the studies included in the systematic review according to AXIS [45]. Individual studies were provided in Supplement 1. All of the studies had a clear aim, appropriate risk factors and outcome variables measured to the aims of the study, discussions and conclusions justified by the results. None of the studies justified their sample sizes. There were only two studies that addressed non-responders [28, 36], and only one study was internally consistent [20].

**Table 5:** Assessment of the studies included in the systematic review according to the appraisal tool for cross-sectional studies (AXIS).

Questions	Yes	No	Uncertain	Not applicable
*Introduction*				
Were the aims/objectives of the study clear?	27	0	0	0
*Methods*				
Was the study design appropriate for the stated aim(s)?	11	16	0	0
Was the sample size justified?	0	27	0	0
Was the target/reference population clearly defined?	19	8	0	0
Was the sample frame taken from an appropriate population base so that it closely represented the target/reference population under investigation?	19	0	8	0
Was the selection process likely to select subjects/participants that were representative of the target/reference population under investigation?	6	16	0	5
Were measures undertaken to address and categorise non-responders?	2	2	0	23
Were the risk factor and outcome variables measured appropriate to the aims of the study?	27	0	0	0
Were the risk factor and outcome variables measured correctly using instruments/measurements that had been trialled, piloted or published previously?	10	17	0	0
Is it clear what was used to determine statistical significance and/or precision estimates?	16	5	0	6
Were the methods (including statistical methods) sufficiently described to enable them to be repeated?	20	7	0	0
*Results*				
Were the basic data adequately described?	26	1	0	0
Does the response rate raise concerns about non-response bias?	0	27	0	0
If appropriate, was information about non-responders described?	2	2	0	23
Were the results internally consistent?	1	25	0	1
Were the results presented for all the analyses described in the methods?	22	5	0	0
*Discussion*				
Were the authors’ discussions and conclusions justified by the results?	27	0	0	0
Were the limitations of the study discussed?	18	9	0	0
*Other*				
Were there any funding sources or conflicts of interest that may affect the authors’ interpretation of the results?	0	9	18	0
Was ethical approval or consent of participants attained?	14	0	13	0

### Inter-agreement reliability

The inter-agreement reliability between the two reviewers was 95.8% (Identification), 92.1% (Screening), 94.2% (Eligibility), and 100% (Inclusion). Each domain provided in AXIS tool had a strong to almost perfect inter-agreement reliability between the independent reviewers, with a kappa score of 0.835 (Introduction), 0.850 (Methods), 0.983 (Results), 0.900 (Discussion), and 0.911 (Other) respectively.

## Discussion

To our knowledge, this is the most up-to-date study to quantitatively outline the possible risks involved in LNI following the surgical extraction of M3M. There was a statistically significant 4.80-fold increase in the risk of temporary LNI when BA+ was used compared to BA-. The incidence of permanent nerve injury was 0.28% with LS compared to 0.18% with BA- and 0.07% with BA+. Most of the articles did not provide sufficient information to develop a risk ratio for permanent injury. Zuniga et al. reported that the incidence of permanent LNI following the removal of M3M to be 0.04–0.60% regardless of the surgical technique [6]. Therefore, the findings in this study are not unusual. Further investigation to determine the incidence of permanent LNI is required so a meta-analysis can be conducted in the future.

Various factors also affected the incidence of LNI. The most commonly found statistically significant risk factors in the literature were angulation of the teeth [16, 19, 20, 26, 46], operator’s experience [17, 20, 25, 26, 29, 46, 47], and the difficulty of extraction (usually measured by the length of operation) [28, 29, 46, 47]. Obiechina and colleagues stated that operating under general anaesthesia may be a risk factor as the tissues can be retracted more, leading to increased stretching causing lingual paraesthesia [34]. Other factors such as sectioning the tooth and removing bone may also increase the risk of LNI [16, 26, 46, 47]. Age is also a suggested risk factor, however, whether or not this was statistically significant varied among the studies [16, 20, 24–26, 29, 36, 47]. The difficulty of extraction can be pre-operatively assessed by taking into account some of these identified risk factors, as well as the operator’s clinical judgement.

A previous systematic review investigated the incidence of LNI following the use of three different techniques, BA-, BA+, LS [12]. Pichler and Beirne found that the incidence of temporary LNI was higher when LS was used versus BA+ when they were compared to BA- (RR—13.3 and 8.8 respectively). Permanent LNI was found to be the lowest when LS was used (0.10%) versus BA+ (0.60%), and BA- (0.20%) [12]. Rapaport and Brown also examined the LNI risk but grouped together all procedures that used a lingual retractor [13]. They determined that when a purpose-built instrument was used to retract lingually, the percentage risk of temporary LNI was lower versus no lingual retraction [0.56% vs 0.60% respectively]. Interesting to note is that the percentage risk of temporary LNI was 7.78% when a non-purpose built instrument was used. The risk of permanent LNI was 0.00%, 0.08% and 0.41% for purpose-built lingual retraction, no lingual retraction and non-purpose built lingual retraction, respectively. Our study did not separate different types of retractors.

A previous Cochrane review was conducted in 2020 analysing techniques for the removal of M3M [48]. This study found that the Peto Odds Ratio of permanent lingual nerve injury was 0.14 [0.00, 6.82] when comparing using a lingual retractor compared to not using one. However, this result was based on 1 study. The Odds ratio in the same Cochrane review for temporary lingual nerve injury when comparing the use of a lingual retractor with no lingual retractor was 4.18 [1.75, 9.98]. However, this was also only based on the results of 3 studies [48].

Our study showed that there was a statistically significant increased risk of temporary LNI when using BA+ versus BA- (RR = 4.80). The prevalence of temporary LNI following BA-, BA+ and LS was found to be 1.24%, 2.39% and 2.44% respectively. This result was consistent with Pichler and Beirne. Their risk ratio when comparing BA+ and BA- was 8.8 and the prevalence of LNI was 0.60% (BA-), 6.40% (BA+), and 9.60% (LS). In both our results and Pichler and Beirne’s, LS had the highest incidence of temporary LNI followed by BA+ and then BA-. However, the prevalence of BA- found by Pichler and Beirne was lower while BA+ and LS were higher [12].

The prevalence of permanent LNI following BA-, BA+ and LS was 0.18%, 0.07%, and 0.28% respectively. There is an increased prevalence of permanent LNI following the use of LS compared to both BA- and BA+. These results were inconsistent with Pichler and Beirne who found that LS had the lowest prevalence of permanent LNI while BA+ had the highest [0.2% (BA-), 0.6% (BA+), 0.1% (LS)] [12]. This discrepancy may be due to insufficient data being available regarding permanent LNI.

Our study had negligible statistical heterogeneity. Quantifying statistical heterogeneity can only be validated if there is an unknown clinical heterogeneity [49]. According to the overall RR in this study, it is most likely that there was no evidence of clinical heterogeneity. Furthermore, according to our systematic review, clinical covariates across all studies (e.g. patient level, intervention level, outcome level) are shown to be similar. This was as a result of the stringent criterion placed to minimise heterogeneity [50]. Furthermore, during the derivation of the forest plot, implementing both fixed- and random- effects model made little difference to the I^2^ value.

### Limitations

One of the major limitations of this systematic review and meta-analysis was that we were unable to calculate the risk ratio for permanent LNI due to the lack of published data. Furthermore, only the RR between BA+ versus BA- was calculated as there was insufficient data to calculate for LS to draw a statistically meaningful conclusion. Another limitation was the exclusion of grey literature from our study; however, the inclusion of grey literature may have further skewed our results due to the lack of peer review within these studies. In addition, many studies were not randomised controlled trials. In many studies, the use of a lingual retractor was used at the clinician’s discretion. This could introduce a level of bias as it may indicate that lingual retraction was used in more difficult cases that were at higher risk of LNI regardless of whether lingual retraction was used or not. Another limitation was that the type of lingual retractor used varied between the articles and some articles did not specify what was used (Table 2). This may introduce a level of bias as Rapaport and Brown found that the percentage risk of permanent LNI was lowest when purpose-built lingual retractors were used [13]. They also found that the use of repurposed lingual retractors had the highest risk of temporary and permanent LNI. Another limitation was the position and impaction of the M3M were not factored in when comparing the incidence of LNI. The position of the M3M has been noted to affect the risk of LNI [51]. However, we were unable to analyse this factor as the studies included in our systematic review did not outline the impaction of the M3M. This could be something that could potentially be explored in the future. Lastly, limitations were also present during data input. When papers did not list the number of subjects or teeth, we adopted a 1:1 ratio for subject:patient when inputting the data. This could potentially skew the results if studies had one patient undergoing two M3M surgical extractions.

### Implication of practice

The global prevalence of impacted M3M is approximately 24% [52]. In 2008, an estimated number of hospitalisations for extraction of impacted M3M in Australia was 97,949 [15–34 years]. This resulted in the total cost of $531 million AUD [53]. In addition, in the United States, approximately 10 million M3M were extracted annually with costs approximating $3 billion USD [54]. One of the risks of M3M is LNI which can negatively impact a patient’s QoL [1], and therefore operators should attempt to minimise the risk as much as possible. Previous studies have used lingual retraction to protect the lingual nerve from injury. As there are limited studies that published the outcomes of permanent LNI, further research is required to assess and analyse the incidence of permanent LNI following these techniques (BA-, BA+, and LS).

## Conclusion

This study has shown that there is a quantifiable increased risk of temporary LNI following the surgical extraction of M3M when BA+ and LS is used compared to BA-. The current literature showed that there was a lower incidence of permanent LNI after using BA+ compared to BA- and LS. LS had a higher temporary and permanent risk for LNI. There is insufficient evidence to determine whether there is a significant advantage of lingual nerve retraction for reducing the risk of permanent LNI despite the low incidence. Larger scale studies are needed to consolidate the findings. Operators should use BA+ and LS with caution due to the increased risk of temporary LNI.

## Supporting information

S1 Checklist PRISMA 2020 checklist. (DOCX)

## Abbreviations

BA-: Buccal approach without lingual flap retraction
BA+: buccal approach with lingual flap retraction
LN: Lingual nerve
LNI: Lingual nerve injury
LS: Lingual split technique
M3M: Mandibular third molar
